# Transcutaneous auricular vagus nerve stimulation and emotional responding in borderline personality disorder: a randomized single-blind, sham-controlled study

**DOI:** 10.1186/s40479-026-00367-x

**Published:** 2026-08-04

**Authors:** Giuseppe Guerriero, Anthony C. Ruocco, Hanne K. Carlsen, Alexander R. Daros, Duaa Kosara, Steinn Steingrimsson

**Affiliations:** 1 Department of Psychiatry and Neurochemistry, Institute of Neuroscience and Physiology, Sahlgrenska Academy, University of Gothenburg, Blå stråket 15, Gothenburg, 41345 Sweden; 2 Research & Development Unit, Lillakliniken Psykiatri Cura, Gothenburg, Sweden; 3 Psychiatry, Aalborg University Hospital, Aalborg, Denmark; 4 Department of Psychological Clinical Science, University of Toronto, Toronto, Canada; 5 Department of Psychology, University of Toronto Scarborough, Toronto, Canada; 6 Department of Psychology, University of Toronto, Toronto, Canada; 7 Department of Psychology, University of Windsor, Windsor, Canada; 8 Department of Psychiatry for Affective Disorders, Sahlgrenska University Hospital, Region Västra Götaland, Gothenburg, Sweden

**Keywords:** Borderline personality disorder, Transcutaneous auricular vagus nerve stimulation, Emotional vulnerability, Negative affect, Interoception, Randomized controlled trial

## Abstract

**Background:**

Borderline personality disorder (BPD) is characterized by severe emotional vulnerability, including heightened sensitivity, exaggerated reactivity, and delayed recovery from emotional arousal. Altered interoceptive-affective processing may contribute to these features. Transcutaneous auricular vagus nerve stimulation (taVNS) provides a non-invasive method for modulating vagal-afferent pathways involved in interoception and emotion regulation. This study examined whether active taVNS, relative to sham stimulation, was associated with lower self-reported negative affect during a video-based mood-induction paradigm in women with BPD.

**Methods:**

Thirty-four female psychiatric outpatients with DSM-5 BPD were randomized to receive active taVNS or sham stimulation during a single-session experimental paradigm. Self-reported negative affect was assessed repeatedly using the Positive and Negative Affect Schedule (PANAS) Negative Affect scale. The primary analysis modeled PANAS Negative Affect across four collapsed task phases: Pre-Induction, Post-Neutral, Post-Negative, and Recovery-Negative, adjusting for baseline negative affect and stimulation intensity. Exploratory physiological analyses examined heart rate, lnRMSSD, phasic electrodermal activity (EDA) response frequency, and tonic skin conductance level.

**Results:**

Active taVNS was associated with lower PANAS Negative Affect across the mood-induction paradigm compared with sham stimulation, Treatment Group: F(1, 36.44) = 5.74, *p* = 0.022. Negative affect varied significantly across task phases, F(3, 94.12) = 22.60, *p* < 0.001, confirming successful mood induction. The Treatment Group × Phase interaction was not significant, F(3, 94.12) = 0.75, *p* = 0.525. Exploratory physiological analyses showed no treatment-related effects on heart rate, lnRMSSD, or phasic EDA response frequency. Tonic skin conductance level showed no main effect of Treatment Group, but an exploratory Treatment Group × Epoch interaction.

**Conclusions:**

In this preliminary single-session randomized sham-controlled study, active taVNS was associated with lower self-reported negative affect during a mood-induction paradigm in women with BPD. This subjective effect was not accompanied by treatment-related effects on heart rate, lnRMSSD, or phasic EDA response frequency, while the tonic skin conductance interaction should be interpreted cautiously as exploratory. These findings support further investigation of taVNS as a mechanistic probe of acute affective responding in BPD.

**Trial registration:**

ClinicalTrials.gov NCT05892900. Retrospectively registered 7 June 2023.

**Supplementary Information:**

The online version contains supplementary material available at 10.1186/s40479-026-00367-x.

## Introduction

Borderline personality disorder (BPD) is characterized by marked affective instability, chronic impulsivity, and recurrent self-destructive behaviours [1, 2]. Although psychotherapeutic approaches such as Dialectical Behaviour Therapy are effective, access remains limited, and there is no clear first-line pharmacological option [3]. Moreover, many patients continue to experience severe emotional distress and recurrent dysregulation despite treatment, suggesting that important biological mechanisms underlying emotional vulnerability remain insufficiently understood [4–6].

According to Linehan’s biosocial theory, BPD is fundamentally a disorder of emotion regulation in which a biologically based emotional vulnerability interacts with invalidating environments [7, 8]. This vulnerability is commonly described as comprising three interrelated features: heightened emotional sensitivity, exaggerated emotional reactivity, and a delayed return to baseline [7, 8]. Although this tripartite description has had major clinical value, the physiological mechanism through which these features emerge, and whether they arise from a single underlying disturbance or from partially independent processes, remains incompletely specified [9, 10].

One plausible candidate mechanism is interoception, broadly defined as the sensing, representation, and interpretation of signals originating within the body [11–13]. Contemporary models of emotion increasingly emphasize that emotional experience depends not only on the magnitude of physiological change, but also on how bodily signals are detected, weighted, and integrated with context and expectations [11, 14–16]. From this perspective, affective instability may arise not only from heightened autonomic activation, but from altered access to, or interpretation of, bodily signals.

This possibility is particularly relevant in BPD. Interoception has been proposed as a potentially important mechanism underlying disturbances in self-processing, emotion processing, and psychosocial functioning in BPD, suggesting that altered bodily signal processing may contribute to core features of the disorder [17]. Preliminary evidence further suggests that early life stress may adversely affect central interoceptive representation and thereby contribute to emotional dysregulation in BPD [17].

Prior work also suggests that individuals with BPD show alterations in autonomic and interoceptive processing, including reduced heart rate variability (HRV), elevated resting arousal in some studies, and differences in cortical representation of bodily signals [18–20]. Neurophysiological findings involving heartbeat-evoked potentials and neuroimaging studies implicating the insula, amygdala, anterior cingulate cortex, and prefrontal regions support the view that emotional dysregulation in BPD may involve altered processing of internal bodily states rather than only heightened peripheral arousal [21–24]. At the same time, the literature remains heterogeneous, and physiological hyperreactivity has not been consistently demonstrated [9, 25–29]. This inconsistency raises the possibility that the core disturbance in emotional vulnerability may lie less in the magnitude of peripheral autonomic responses per se and more in the threshold at which such responses become affectively salient.

Drawing on contemporary interoceptive accounts of emotion [11, 14–16], we use an interoceptive-threshold framework as a conceptual rationale for the present study. In this framework, emotional vulnerability in BPD may partly reflect a lowered threshold at which bodily/interoceptive signals become affectively salient. Such a threshold shift could contribute to heightened emotional sensitivity, stronger subjective reactivity, and slower subjective recovery, without necessarily requiring exaggerated peripheral autonomic output at every stage [11, 14–16]. taVNS provides a non-invasive method for modulating afferent input from the auricular branch of the vagus nerve to brainstem nuclei, including the nucleus tractus solitarius, with downstream projections to central autonomic and interoceptive-affective networks involving the insula, cingulate, prefrontal, and limbic regions [30–35]. Through these pathways, taVNS may influence how bodily signals are integrated with emotional appraisal and regulation, consistent with evidence that taVNS can modulate cortical interoceptive processing and emotion-regulation-related responses [36–39]. However, effects of taVNS on peripheral autonomic indices such as heart rate variability remain mixed [40, 41], and the present study did not measure central interoceptive processing directly. The framework is therefore used here only as a conceptual rationale for examining whether vagal-afferent modulation is associated with acute changes in subjective emotional responding during mood induction.

Recent work in healthy participants suggests that taVNS may influence interoceptive processing, including interoceptive accuracy during heartbeat detection tasks and modulation of heartbeat-evoked potentials localized to insular, somatosensory, and prefrontal regions implicated in the cortical representation of bodily signals [31, 38, 39]. Together, these findings support the use of taVNS as an experimental probe for examining whether modulation of vagal-afferent signalling is associated with altered emotional responding.

Experimental studies, mostly in healthy participants or non-BPD samples, further suggest that taVNS can influence emotion recognition, mood, and stress-related processes, although findings regarding autonomic effects remain mixed [35, 41–46]. Some studies have reported improved emotion recognition or mood-related outcomes [42–45], whereas meta-analytic evidence indicates that group-level effects of active versus sham taVNS on vagally mediated heart rate variability are inconsistent [41]. These observations suggest that taVNS-related changes in affective responding may not necessarily be accompanied by a simple or uniform increase in peripheral parasympathetic activity.

To our knowledge, taVNS has not previously been investigated in patients with BPD. The present single-session study was designed to test whether acute vagal-afferent modulation is associated with reduced subjective negative affect during emotional challenge. This acute experimental approach is distinct from repeated-session neuromodulation protocols aimed at inducing longer-lasting clinical or network-level changes [47]. Rather, it was intended to examine whether active taVNS, relative to sham stimulation, can influence emotional experience while stimulation is ongoing, consistent with the possibility that vagal-afferent input may acutely modulate interoceptive-affective processing.

The registered primary outcome was post-induction negative emotional arousal, assessed using the Positive and Negative Affect Schedule (PANAS) Negative Affect scale [48] immediately after mood induction. In the present analysis, the registered PANAS outcomes were modeled jointly across four collapsed task phases to provide a parsimonious estimate of treatment-group differences across the paradigm while reducing model multiplicity. We hypothesized that active taVNS would be associated with lower self-reported negative affect compared with sham stimulation. A global downward shift in negative affect across phases, rather than a phase-specific effect confined to a single component of emotional responding, was considered compatible with the view that taVNS may influence a common underlying process linked to emotional vulnerability. Secondary exploratory analyses examined whether treatment-related differences were also observed in peripheral autonomic indices of cardiac and electrodermal activity.

## Material and methods

### Study design and participants

This study reports data from a randomized, single-blind, sham-controlled trial examining the acute effects of transcutaneous auricular vagus nerve stimulation (taVNS) on emotional responding in BPD. A detailed study protocol has been published previously [49]. The experiment was conducted as a single-session laboratory study in Sweden and retrospectively registered at ClinicalTrials.gov (NCT05892900) on 7 June 2023 [49]. The study was conducted in accordance with the Declaration of Helsinki and was approved by the Swedish Ethical Review Authority (Etikprövningsmyndigheten; Dnr 2022–05841-01). A subsequent amendment was also approved by the Swedish Ethical Review Authority (Dnr 2023–06370-02). Participants were female psychiatric outpatients aged 18–50 years with a current diagnosis of BPD according to the Diagnostic and Statistical Manual of Mental Disorders, Fifth Edition (DSM-5) [1]. Recruitment took place through psychiatric services in Sweden, including referrals from treating clinicians and voluntary response to on-site advertisements. Interested individuals underwent an initial telephone screening and, if potentially eligible, were invited to the laboratory, where written informed consent was obtained before any study procedures were performed. Participants did not receive financial compensation for taking part in the study.

BPD diagnosis was confirmed using the Structured Clinical Interview for DSM-5 Personality Disorders (SCID-5-PD) [50]. The Mini International Neuropsychiatric Interview (MINI) version 7.0.0 [51] was used during screening to assess exclusionary psychiatric conditions. Diagnostic assessments and study procedures were conducted by medical doctors with clinical experience in structured diagnostic interviewing. Inclusion criteria were Swedish language proficiency, ability to provide informed consent, female sex, age between 18 and 50 years, current DSM-5 BPD [1], and ability to comply with study procedures. Exclusion criteria included unstable medical or neurological illness, pregnancy, significant neurological disorder, delirium or dementia, autism spectrum disorder or attention-deficit/hyperactivity disorder, clinically significant uncorrectable sensory impairment, current or recent alcohol or substance use disorder, daily treatment with antiepileptic drugs or benzodiazepines, intracranial implants or non-removable metal objects near the head, and a lifetime diagnosis of bipolar disorder or chronic psychotic disorder.

The planned target sample was 42 participants, corresponding to 21 participants per arm. This target included an expected attrition allowance of approximately 20%; the minimum sample size required by the original power calculation was 34 participants. In total, 43 participants were assessed for eligibility, of whom 9 were excluded before randomization because they did not meet inclusion criteria or met exclusion criteria. The final randomized sample therefore consisted of 34 participants because only 34 eligible participants were available for randomization within the recruitment period. All randomized participants completed the experimental protocol and were included in the primary self-report analyses. Outcome-specific exclusions were applied to physiological analyses based on signal quality, as described below.

### Randomization and blinding

Participants were randomized in a 1:1 ratio to active taVNS or sham stimulation. The allocation sequence was generated using computerized random number generation by a member of the research team not involved in enrolment or testing and was placed in sealed, opaque, sequentially numbered envelopes. At the time of allocation, the investigator opened the next envelope in sequence. The study used a single-blind design: participants were blinded to treatment allocation, whereas investigators were not. Because both active and sham stimulation produced a perceptible cutaneous sensation, the two conditions were designed to be subjectively similar [49].

### Stimulation procedure

Stimulation was delivered using the tVNS® device (tVNS Technologies GmbH, Germany). In the active condition, the electrode was placed at the left cymba conchae; in the sham condition, the electrode was placed at the centre of the left earlobe, an anatomical site commonly used as a control condition because it is not considered to provide effective vagal stimulation [49, 52]. The device delivered biphasic stimulation at 25 Hz in alternating cycles of 28 s on and 32 s off. Stimulation intensity ranged from 0.1 to 5.0 mA and was individually titrated in increments of 0.1 mA until participants reported a clearly noticeable tingling or pulsating sensation that was not painful or uncomfortable. In the final sample, the mean stimulation intensity was 0.9 ± 0.8 mA. After the individually tolerated stimulation intensity had been reached, stimulation continued for 4 min before the emotional task began. Participants in both groups received identical nominal stimulation parameters, differing only in stimulation site.

### Experimental paradigm

Each study visit lasted approximately 2 h in total. After written informed consent, participants completed screening and baseline procedures, including clinical and anamnestic information, self-report questionnaires, resting pulse and blood pressure, and assessment of recent substance use, including nicotine and caffeine. These measures, including the Borderline Symptom List (BSL-23) [53], Difficulties in Emotion Regulation Scale (DERS) [54], background clinical variables, recent substance use, and resting physiological measures, were collected during the same laboratory visit before the experimental stimulation and mood-induction procedure.

Participants were seated comfortably in front of a computer monitor in an adjustable chair. Before the task, physiological sensors were attached. Continuous electrocardiography (ECG) and Galvanic Skin Response (GSR) recording started immediately before the baseline PANAS assessment and continued throughout the experimental procedure. Participants were informed that the video clips could include emotionally provocative content, including violence and sexual content. They were instructed to maintain visual attention to the monitor, avoid closing their eyes as much as possible, and immerse themselves in each video. The room lights were dimmed to facilitate engagement with the task. An investigator or research assistant remained quietly present in the room but out of the participant’s direct view throughout the procedure.

**Fig. 1:**
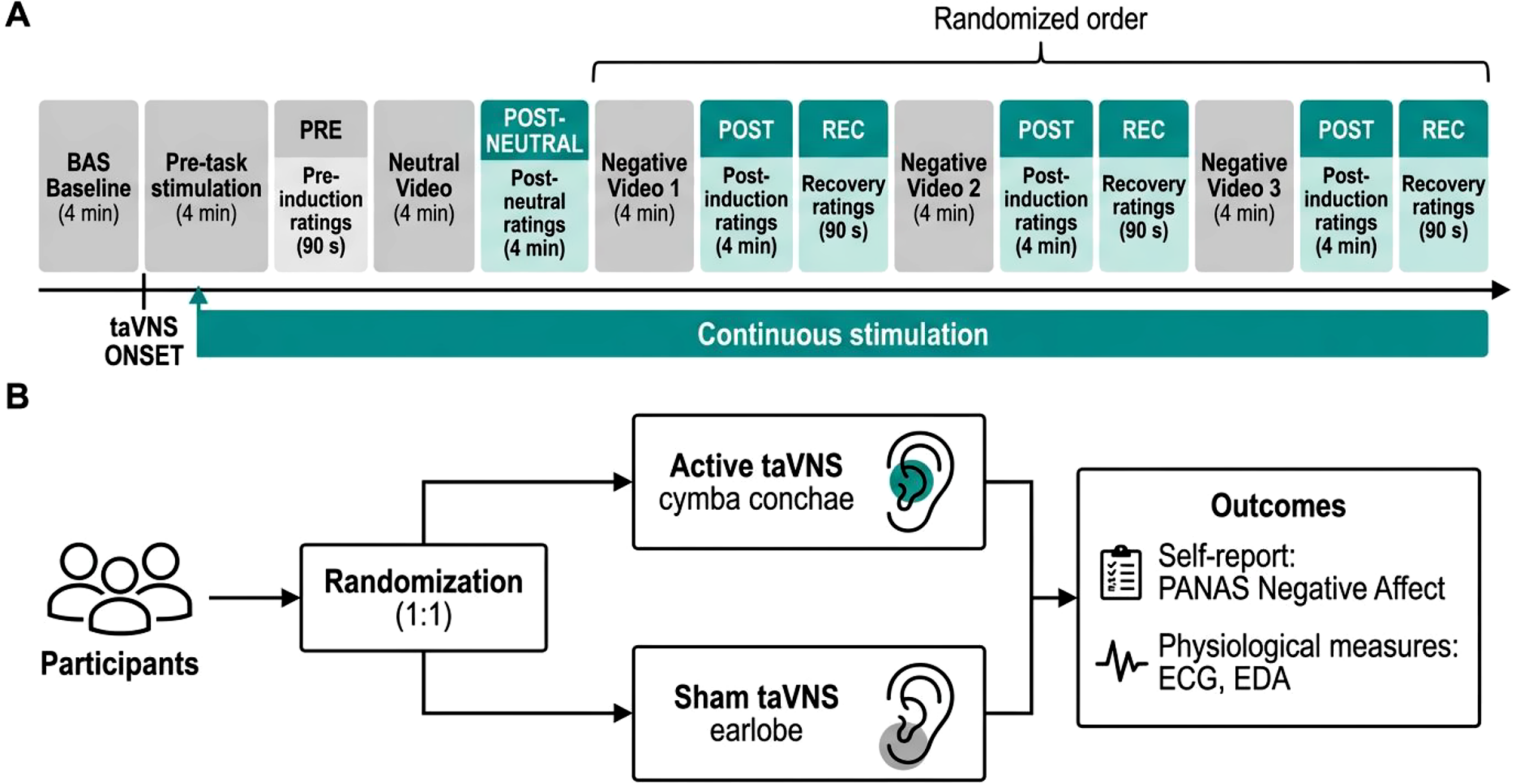
Experimental design and mood-induction paradigm during active or sham taVNS. (**A**) Phase-based experimental timeline. Participants first completed a baseline (BAS) assessment of subjective affect and physiological activity (4 min) prior to stimulation. Active or sham transcutaneous auricular vagus nerve stimulation (taVNS) was then initiated and delivered continuously throughout the remainder of the experimental stimulation protocol, which lasted approximately 45 min. This was followed by a pre-task stimulation period (4 min), after which participants completed pre-induction (PRE) ratings (90 s). Next, participants viewed a neutral video (4 min), followed by a post-neutral rating period (4 min). Subsequently, three negative videos (domestic violence, funeral, and sexual assault), each lasting 4 min, were presented in randomized order. Each negative video was followed by post-induction (POST) ratings (4 min) and a recovery (REC) phase (90 s). No recovery phase followed the neutral video. (**B**) Randomization and outcome measures. Participants were randomly assigned (1:1) to receive either active taVNS applied to the cymba conchae or sham stimulation applied to the earlobe. Outcome measures included self-reported negative affect (PANAS negative affect scale) and physiological recordings obtained via electrocardiography (ECG) and electrodermal activity (EDA)

The experimental stimulation protocol lasted approximately 45 min and consisted of an initial baseline assessment, followed by continuous active or sham stimulation and a standardized mood induction task administered through Qualtrics using fixed timing and automatic page advance (Fig. 1A). Participants were randomized 1:1 to active taVNS of the cymba conchae or sham stimulation of the earlobe, with self-reported PANAS Negative Affect and physiological indices derived from ECG and electrodermal activity (EDA) as outcomes (Fig. 1B).

The mood-induction paradigm was adapted from prior work by Daros and colleagues, with modifications specified in the present trial protocol [49, 55]. In the original study, the procedure was tested in 90 women, including 30 participants with BPD, 30 participants with mixed anxiety/depressive disorders, and 30 healthy controls [55]. The same three negative video themes used in the present study—domestic violence, a funeral scene, and sexual assault—were shown to increase negative mood across groups. Participants with BPD reported higher negative mood than healthy controls at baseline, after the neutral video, after each negative video, and during recovery following the funeral video, with medium-to-large between-group effects (d = 0.52–1.61). Participants with BPD also reported greater subjective difficulty managing emotional responses during the negative videos than healthy controls (d = 0.55–0.80). Group × Time interactions were not significant for any video (ps = 0.34–0.94), suggesting broadly similar trajectories of mood change across groups but higher overall negative mood in BPD. Negative mood decreased significantly from post-induction to recovery after each negative video, with large effects (η^2^ = 0.33–0.54). In the present study, the video paradigm was used as a within-sample mood-induction procedure to elicit negative affect during ongoing active or sham stimulation, rather than to test whether the stimuli produced BPD-specific responses relative to healthy or clinical control groups. The individual clips were not drawn from a single standardized stimulus database with uniform normative valence and arousal ratings.

The paradigm consisted of one neutral and three negative 4-min film clips. The neutral video was presented first, whereas the three negative videos were presented in randomized order using the Qualtrics randomizer function (Fig. 1A). The neutral video was selected to elicit very low levels of emotion and was presented first to orient participants to the procedure. The three negative videos depicted domestic violence, a funeral scene, and sexual assault, respectively [49, 55].

At baseline, participants completed self-reported ratings of affect before stimulation onset. Stimulation was then initiated. After a 4-min pre-task stimulation period without emotional stimuli, participants completed a pre-induction affect rating. The neutral video was then presented, followed by a post-neutral rating period. This was followed by the three negative videos. Each negative video lasted 4 min and was followed by post-video ratings and a subsequent 90-s recovery phase. No recovery phase followed the neutral video [49, 55]. All task elements, including instructions, videos, and rating scales, were administered through the same Qualtrics workflow.

### Measures

The primary outcome for the present report was self-reported negative affect measured repeatedly with the Negative Affect subscale of the PANAS [48]. PANAS ratings were obtained at baseline, after the initial stimulation-only period (Pre-Induction), during the post-neutral rating period (Post-Neutral), after each of the three negative videos (Post-Negative), and after each of the three recovery periods following the negative videos (Recovery-Negative). In the original protocol, these repeated ratings were intended to index different components of emotional responding across the task. In the present report, they were analyzed jointly as repeated observations of negative affect across the experimental paradigm. After each video, participants also rated their perceived difficulty managing emotional responses during video presentation, corresponding to the registered outcome concerning perceived effectiveness or difficulty in managing emotions during the task.

To characterize the clinical sample at baseline, participants also completed the Difficulties in Emotion Regulation Scale (DERS) [54] and the Borderline Symptom List-23 (BSL-23) [53]. For the DERS-36, reverse-scored items were recoded before total scores were computed. When no more than 10% of DERS items were missing, total scores were prorated by multiplying the mean of valid items by 36; otherwise, the total score was treated as missing. Screening additionally included demographic and clinical background variables, concomitant medication use, recent nicotine, caffeine, alcohol, or drug intake, and resting vital signs including pulse and blood pressure. Previously reported and sample-specific internal consistency estimates for the self-report measures are provided in Supplementary Table S1.

Physiological signals were recorded continuously throughout the procedure using the iMotions® Biometric Research Platform (version 10.1; iMotions A/S, Copenhagen, Denmark), integrating Shimmer3 ECG and GSR+ modules at a sampling rate of 1000 Hz. ECG was recorded using a five-electrode configuration placed on the torso according to manufacturer guidance to optimize R-wave detection. EDA was recorded from the volar surface of the fingers of the non-dominant hand using a bipolar electrode configuration. Participants were instructed to remain as still as possible and to avoid unnecessary limb movements during recording. These recordings were collected to complement self-report data and to characterize autonomic responding during the experimental procedure.

### Physiological signal processing

ECG recordings were processed to detect R peaks and derive interbeat intervals (IBIs). The resulting IBI series were visually inspected for implausible intervals and obvious artifacts, and segments with poor signal quality were excluded. Mean heart rate and root mean square of successive differences (RMSSD) were then calculated for each task window, and RMSSD was natural log-transformed prior to statistical analysis to reduce skewness.

Electrodermal activity (EDA)/galvanic skin response (GSR) recordings were processed using the iMotions/Shimmer processing workflow. Phasic EDA response frequency was indexed as peaks per minute. Tonic skin conductance level (SCL) was extracted from the iMotions “Tonic Signal” output. For GSR-based analyses, the same task-defined epochs were used as for the other physiological outcomes: stimulation-alone, neutral-video, and negative-video. The negative-video epoch was computed as the mean of the three negative-video epoch means, so that each negative video contributed equally.

Outcome-specific exclusions were applied based on signal quality. One participant was excluded from ECG-derived analyses because ECG-derived indices were physiologically implausible. One participant was excluded from GSR-based analyses because tonic SCL values were implausibly high and virtually invariant across all extracted task epochs, with no detected phasic electrodermal responses, suggesting signal saturation or processing artifact. Zero values in EDA response frequency were retained as valid observations when the GSR signal was otherwise technically usable, as they indicate absence of detected phasic responses during the epoch rather than missing data.

### Statistical analysis

The registered PANAS-based outcomes included post-induction PANAS Negative Affect as the primary outcome, and pre-induction and recovery PANAS Negative Affect as secondary outcomes. Because these outcomes represented repeated ratings of negative affect within the same emotional challenge paradigm, the primary analysis modeled PANAS Negative Affect jointly across four collapsed task phases: Pre-Induction, Post-Neutral, Post-Negative, and Recovery-Negative. This approach was chosen to reduce model multiplicity, avoid overinterpretation of partially overlapping phase-specific contrasts in a modestly sized sample, and provide a parsimonious estimate of treatment-group differences across the task. We report how we determined our sample size, all data exclusions, all manipulations, and all measures included in the study.

The primary PANAS model included Treatment Group, Phase, and the Treatment Group × Phase interaction as fixed effects, with baseline PANAS Negative Affect and stimulation intensity entered as covariates. Participant ID was specified as the repeated subject variable, and a first-order autoregressive covariance structure [AR(1)] was used to model within-participant dependency across phases. A sensitivity analysis using a compound symmetry covariance structure was also performed. Random slopes for Phase were explored but were not retained because models including random slopes either failed to converge or produced unstable covariance solutions; therefore, the final models used repeated-measures covariance structures.

To maintain transparency with respect to the registered outcomes, outcome-specific supplementary analyses were also conducted. The registered post-induction PANAS outcome was analyzed using a mixed-effects model including Treatment Group, VideoType, Treatment Group × VideoType, baseline PANAS Negative Affect, and stimulation intensity. The registered pre-induction PANAS outcome was analyzed using ANCOVA with Treatment Group, baseline PANAS Negative Affect, and stimulation intensity. The registered recovery PANAS outcome was analyzed using a mixed-effects model including Treatment Group, Time, Treatment Group × Time, VideoType, baseline PANAS Negative Affect, and stimulation intensity, with Time modeled as repeated within participant-by-video. Perceived difficulty managing emotional responses was analyzed using a mixed-effects model including Treatment Group, VideoType, Treatment Group × VideoType, and stimulation intensity. Sensitivity analyses additionally adjusting for VideoOrder were performed for the video-specific post-induction PANAS, recovery PANAS, and perceived difficulty managing emotional responses models.

Exploratory secondary physiological analyses were based on physiological data recorded continuously during the experimental task and summarized within task-defined stimulation and video-viewing epochs, rather than within post-stimulus rating periods. The analyzed epochs were stimulation-alone, neutral-video, and negative-video. The negative-video epoch was computed as the mean across the three 4-min negative video segments, so that each negative video contributed equally. Baseline physiology was used as a covariate rather than as a repeated outcome, because the baseline period also involved completion of baseline affect ratings and was therefore considered less suitable as a task-free physiological comparison epoch. Separate mixed-effects models were estimated for heart rate, lnRMSSD, phasic EDA response frequency indexed as peaks per minute, and tonic SCL. Each model included Treatment Group, Epoch, and the Treatment Group × Epoch interaction as fixed effects, with the corresponding baseline physiological value, stimulation intensity, and recent nicotine use entered as covariates. Recent nicotine use was defined as any nicotine use during the 12 h preceding the experiment. Outcome-specific exclusions were applied according to signal quality. ECG-derived analyses excluded one participant with invalid ECG-derived indices. GSR-based analyses excluded one participant because of implausibly high and invariant tonic SCL values with no detected phasic electrodermal responses, suggesting signal saturation or processing artifact.

Given the potential relevance of nicotine use for arousal and sensory/interoceptive processing, the primary PANAS model was additionally rerun in a sensitivity analysis including recent nicotine use as a covariate. This analysis was not considered the primary model but was used to examine the robustness of the main PANAS finding.

All analyses were performed in SPSS Statistics version 30.0 using two-tailed tests with *p* < 0.05.

### Use of AI tools

During the preparation of this manuscript, ChatGPT (OpenAI) was used to support language editing, improve clarity of expression, and assist with restructuring text. FigureLabs and Julius AI were used to support the preparation and refinement of figures and graphical presentation. All statistical analyses, study design decisions, interpretation of results, and scientific conclusions were conducted, verified, and approved by the authors.

## Results

### Participant flow and baseline characteristics

**Fig. 2:**
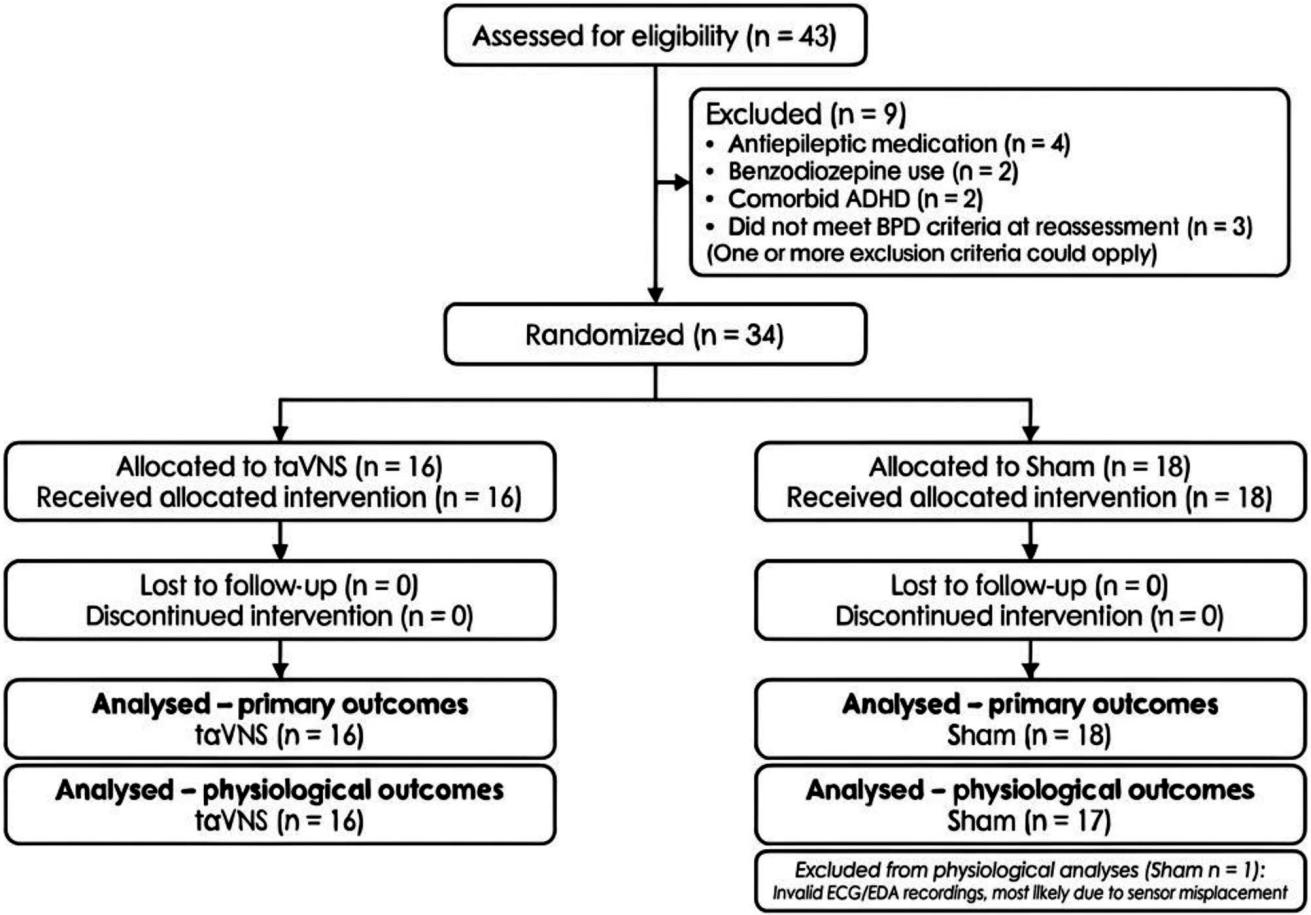
CONSORT flow diagram of participant recruitment, randomization, and analysis. Of the 43 individuals assessed for eligibility, 9 were excluded before randomization because they met one or more exclusion criteria or did not meet BPD criteria at reassessment. Thirty-four participants were randomized to receive either active taVNS (*n* = 16) or sham stimulation (*n* = 18). All randomized participants received the allocated intervention, completed the experimental protocol, and were included in the primary self-report analyses. Outcome-specific exclusions were applied to physiological analyses based on signal quality: one participant was excluded from ECG-derived analyses because of invalid ECG-derived indices, and one participant was excluded from GSR-based analyses because of implausibly high and invariant tonic SCL values with no detected phasic electrodermal responses, suggesting signal saturation or processing artifact

Participant flow is shown in Fig. 2. Thirty-four participants were randomized and included in the primary PANAS analyses: 16 participants in the active taVNS group and 18 participants in the sham stimulation group. No participants were excluded from the self-report analyses after randomization. The discrepancy between the planned target sample and the final randomized sample, as well as outcome-specific exclusions for physiological analyses, are described in the Methods and shown in the CONSORT flow diagram. No serious adverse events occurred during the experimental session, and no participant discontinued the procedure because of stimulation-related discomfort.

Baseline demographic, clinical, stimulation-related, and physiological characteristics are summarized descriptively in Table 1.

**Table 1:** Baseline demographic, clinical, stimulation-related, and physiological characteristics of the participants

Variables	Active taVNS (n = 16)	Sham stimulation (n = 18)
	**Mean (SD) or n (%)**	**Mean (SD) or n (%)**
Demographic		
Age, years	31.6 (8.6)	32.7 (9.5)
Clinical and physiological parameters		
Height, cm	168.8 (5.3)	164.7 (5.8)
Weight, kg	84.8 (28.7)	74.9 (30.7)
Pulse, bpm	72.8 (10.7)	80.0 (16.7)
Blood pressure – diastolic, mmHg	86.0 (11.1)	84.8 (15.4)
Blood pressure – systolic, mmHg	125.3 (10.9)	120.5 (14.1)
Assessment scales – baseline		
Difficulties in Emotion Regulation Scale (DERS), total score	132.13 (21.84)	121.13 (17.03)
Borderline Symptom List-23 (BSL-23), mean score	2.3 (0.8)	2.0 (0.7)
Positive and Negative Affect Schedule, Negative Affect (PANAS-NA), total score	20.1 (7.5)	19.4 (6.4)
Stimulation parameters		
Stimulation intensity, mA	1.12 (1.05)	0.75 (0.26)
Electrophysiology – baseline		
Heart rate, average bpm	78.3 (10.9)	81.4 (13.7)
Heart rate variability (HRV), lnRMSSD	3.47 (0.63)	3.47 (0.77)
Electrodermal activity (EDA), peaks/min	5.0 (3.9)	2.5 (2.8)
Electrodermal activity (EDA), tonic level	2.8 (2.0)	1.8 (1.7)
Current psychiatric comorbidities		
Posttraumatic stress disorder (PTSD)	7 (43.8)	6 (33.3)
Eating disorder (ED)	0 (0.0)	1 (5.6)
Major depression (MD)	1 (6.3)	2 (11.1)
Generalized anxiety disorder (GAD)	1 (6.3)	3 (16.7)
Panic disorder (PD)	0 (0.0)	2 (11.1)
Current medications		
Antidepressants	11 (68.8)	11 (61.1)
Antipsychotics	5 (31.3)	2 (11.1)
Sedating/tranquilizing agents (non-BDZ)	3 (18.8)	8 (44.4)
Vitamin/herbal supplements	0 (0.0)	2 (11.1)
Analgesics/anti-inflammatory medication	0 (0.0)	1 (5.6)
Other medications	8 (50.0)	8 (44.4)
Substances used before the procedure		
Nicotine	6 (37.5)	6 (33.3)
Alcohol/drug use	0 (0.0)	0 (0.0)
Coffee	8 (50.0)	6 (33.3)

**Abbreviations:** BDZ, benzodiazepines; bpm, beats per minute; EDA, electrodermal activity; HRV, heart rate variability; lnRMSSD, natural logarithm of the root mean square of successive differences; PANAS-NA, Positive and Negative Affect Schedule Negative Affect; SCL, skin conductance level; SD, standard deviation; taVNS, transcutaneous auricular vagus nerve stimulation

**Note:** Baseline characteristics are presented descriptively in accordance with CONSORT recommendations. Physiological baseline variables are reported for the corresponding physiological analysis samples; outcome-specific exclusions are described in the Methods and shown in the CONSORT flow diagram

### Self-reported negative affect across the emotional challenge paradigm

The primary mixed-effects model examined PANAS Negative Affect across four collapsed task phases: Pre-Induction, Post-Neutral, Post-Negative, and Recovery-Negative. The model included Treatment Group, Phase, and the Treatment Group × Phase interaction as fixed effects, with baseline PANAS Negative Affect and stimulation intensity entered as covariates. The primary mixed-effects model results are shown in Table 2.

**Table 2:** Primary mixed-effects model for PANAS negative affect across the emotional challenge paradigm

Effect	Active taVNS adjusted mean [95% CI]	Sham stimulation adjusted mean [95% CI]	Adjusted difference active - sham [95% CI]	F(df1, df2)	p	Approx. partial η^2^	Interpretation
Treatment Group	17.25 [14.85, 19.66]	21.22 [18.96, 23.49]	−3.97 [−7.33, −0.61]	F(1, 36.44) = 5.74	0.022	0.14	Active taVNS was associated with lower PANAS Negative Affect than sham stimulation
Phase	—	—	—	F(3, 94.12) = 22.60	<0.001	0.42	PANAS Negative Affect varied across task phases
Treatment Group × Phase	—	—	—	F(3, 94.12) = 0.75	0.525	0.02	No evidence of a phase-specific treatment effect
Baseline PANAS Negative Affect	—	—	—	F(1, 36.71) = 17.75	<0.001	0.33	Baseline PANAS Negative Affect predicted subsequent ratings
Stimulation intensity	—	—	—	F(1, 36.71) = 0.01	0.941	<0.01	Stimulation intensity was not associated with PANAS Negative Affect

**Note:** PANAS = Positive and Negative Affect Schedule. The model included Treatment Group, Phase, Treatment Group × Phase, baseline PANAS Negative Affect, and stimulation intensity. Participant ID was specified as the repeated subject variable, and a first-order autoregressive covariance structure [AR(1)] was used to model within-participant dependency across phases. Adjusted means are estimated marginal means. The adjusted difference is shown as active taVNS minus sham stimulation. Overall phase-specific estimated marginal means were: Pre-Induction = 16.52 [14.49, 18.56], Post-Neutral = 17.80 [15.77, 19.83], Post-Negative = 23.70 [21.67, 25.73], and Recovery-Negative = 18.94 [16.91, 20.97]. Approximate partial η^2^ values were calculated from the F statistic and denominator degrees of freedom

Active taVNS was associated with lower self-reported negative affect across the emotional challenge paradigm compared with sham stimulation, Treatment Group: F(1, 36.44) = 5.74, *p* = 0.022. There was also a significant main effect of Phase, F(3, 94.12) = 22.60, *p* < 0.001, whereas the Treatment Group × Phase interaction was not significant, F(3, 94.12) = 0.75, *p* = 0.525. Baseline PANAS Negative Affect significantly predicted subsequent PANAS Negative Affect ratings, F(1, 36.71) = 17.75, *p* < 0.001, whereas stimulation intensity was not associated with PANAS Negative Affect, F(1, 36.71) = 0.01, *p* = 0.941.

**Fig. 3:**
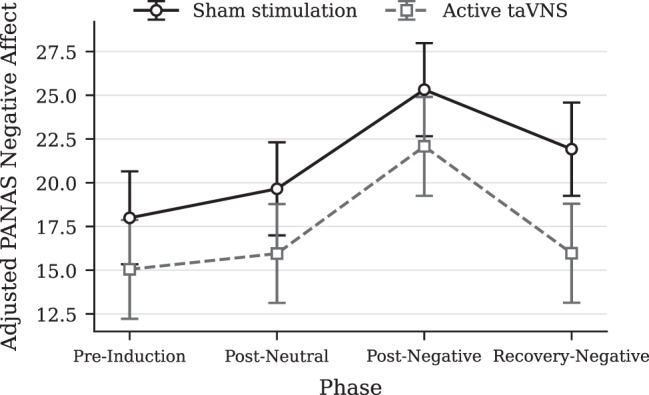
Adjusted PANAS negative affect across the emotional challenge paradigm by treatment group. Values represent estimated marginal means with 95% confidence intervals from the primary mixed-effects model adjusted for baseline PANAS negative affect and stimulation intensity. PANAS negative affect increased after the negative-affect video blocks and decreased during recovery. Across phases, active taVNS was associated with lower PANAS negative affect than sham stimulation, whereas the Treatment Group × Phase interaction was not significant. PANAS = Positive and negative affect Schedule; taVNS = transcutaneous auricular vagus nerve stimulation

Adjusted marginal means showed lower overall PANAS Negative Affect in the active taVNS group than in the sham group (17.25, 95% CI 14.85–19.66 vs. 21.22, 95% CI 18.96–23.49). Across both groups, PANAS Negative Affect was lowest during Pre-Induction (16.52, 95% CI 14.49–18.56), increased slightly after the neutral video (Post-Neutral: 17.80, 95% CI 15.77–19.83), peaked after the negative video blocks (Post-Negative: 23.70, 95% CI 21.67–25.73), and decreased during Recovery-Negative (18.94, 95% CI 16.91–20.97). These adjusted marginal means are illustrated in Fig. 3.

As a manipulation check, pairwise comparisons confirmed that PANAS Negative Affect was significantly higher during Post-Negative than during Pre-Induction, Post-Neutral, and Recovery-Negative, all *p* < 0.001. This pattern indicates that the negative video blocks successfully induced self-reported negative affect.

### Registered outcome-specific analyses

The relationship between prespecified outcomes and the analytic implementation is summarized in Supplementary Table S2. Registered outcome-specific analyses are reported in Supplementary Table S3. In brief, the registered post-induction PANAS analysis showed lower adjusted post-video negative affect in the active taVNS group than in the sham group, although this difference did not reach statistical significance, Treatment Group: F(1, 34.50) = 2.46, *p* = 0.126. There was a significant main effect of VideoType, F(3, 90.07) = 57.33, *p* < 0.001, indicating that the video clips differed in their affective impact, whereas the Treatment Group × VideoType interaction was not significant, F(3, 90.07) = 0.53, *p* = 0.664.

The registered pre-induction PANAS analysis showed significantly lower PANAS Negative Affect in the active taVNS group than in the sham group after adjustment for baseline PANAS Negative Affect and stimulation intensity, Treatment Group: F(1, 30) = 4.50, *p* = 0.042. The recovery analysis showed a significant Treatment Group × Time interaction, F(1, 99.00) = 4.30, *p* = 0.041, indicating a larger decrease from post-induction to recovery in the active taVNS group than in the sham group. Perceived difficulty in managing emotional responses during video presentation (PEME) did not differ between treatment groups, Treatment Group: F(1, 38.81) = 0.03, *p* = 0.868, although there was a significant main effect of VideoType, F(3, 90.59) = 21.35, *p* < 0.001.

### Physiological outcomes

Exploratory secondary physiological analyses were conducted for heart rate, lnRMSSD, phasic EDA response frequency indexed as peaks per minute, and tonic SCL. These models used task-defined stimulation and video-viewing epochs: stimulation-alone, neutral-video, and negative-video. The negative-video epoch was computed as the mean across the three 4-min negative video segments. Baseline physiology was included as a covariate in each model, together with stimulation intensity and recent nicotine use. Full exploratory physiological model results are reported in Supplementary Table S4A, and epoch- and treatment-group-specific estimated marginal means are reported in Supplementary Table S4B.

For heart rate, there was no significant effect of Treatment Group, F(1, 29.38) = 1.32, *p* = 0.260, no significant effect of Epoch, F(2, 60.44) = 0.73, *p* = 0.485, and no significant Treatment Group × Epoch interaction, F(2, 60.44) = 0.04, *p* = 0.965. Baseline heart rate was a strong predictor of task heart rate, F(1, 29.45) = 462.25, *p* < 0.001, whereas stimulation intensity, F(1, 29.45) = 0.24, *p* = 0.631, and recent nicotine use, F(1, 29.45) = 0.12, *p* = 0.736, were not significant predictors.

For lnRMSSD, there was no significant effect of Treatment Group, F(1, 33.69) = 1.84, *p* = 0.184, no significant effect of Epoch, F(2, 64.15) = 0.86, *p* = 0.430, and no significant Treatment Group × Epoch interaction, F(2, 64.15) = 0.41, *p* = 0.663. Baseline lnRMSSD significantly predicted task lnRMSSD, F(1, 34.03) = 123.69, *p* < 0.001, whereas stimulation intensity, F(1, 34.03) = 0.53, *p* = 0.471, and recent nicotine use, F(1, 34.03) = 1.38, *p* = 0.248, were not significant predictors.

For phasic EDA response frequency, there was no significant effect of Treatment Group, F(1, 31.62) = 1.75, *p* = 0.195, no significant effect of Epoch, F(2, 61.91) = 0.95, *p* = 0.393, and no significant Treatment Group × Epoch interaction, F(2, 61.91) = 0.06, *p* = 0.945. Baseline peaks per minute significantly predicted task peaks per minute, F(1, 32.07) = 99.67, *p* < 0.001, whereas stimulation intensity, F(1, 32.07) = 0.34, *p* = 0.562, and recent nicotine use, F(1, 32.07) = 0.92, *p* = 0.344, were not significant predictors.

For tonic SCL, there was no significant main effect of Treatment Group, F(1, 28.07) = 0.41, *p* = 0.525. However, there was a significant main effect of Epoch, F(2, 61.38) = 4.59, *p* = 0.014, and a significant Treatment Group × Epoch interaction, F(2, 61.38) = 4.86, *p* = 0.011. Baseline tonic SCL significantly predicted task tonic SCL, F(1, 27.73) = 206.36, *p* < 0.001, whereas stimulation intensity, F(1, 27.73) = 0.32, *p* = 0.578, and recent nicotine use, F(1, 27.73) = 1.97, *p* = 0.172, were not significant predictors. Estimated marginal means indicated that tonic SCL remained relatively stable across epochs in the sham group, whereas the active taVNS group showed higher tonic SCL during the negative-video epoch compared with the neutral-video and stimulation-alone epochs.

Taken together, the exploratory physiological analyses did not provide evidence that active taVNS modulated heart rate, lnRMSSD, or phasic EDA response frequency relative to sham stimulation. Tonic SCL showed a significant epoch-related Treatment Group × Epoch interaction, driven by higher tonic SCL during the negative-video epoch in the active taVNS group. Given the exploratory nature of the physiological analyses, this finding should be interpreted cautiously.

### Sensitivity analyses

A sensitivity analysis of the primary PANAS model using a compound symmetry covariance structure yielded the same pattern of findings, with a significant main effect of Treatment Group, F(1, 30) = 4.79, *p* = 0.036, a significant main effect of Phase, F(3, 96) = 19.55, *p* < 0.001, and no significant Treatment Group × Phase interaction, F(3, 96) = 0.78, *p* = 0.507.

Given the potential relevance of nicotine use for arousal and sensory/interoceptive processing, the primary PANAS model was additionally rerun with recent nicotine use as a covariate. This analysis yielded the same pattern of findings: active taVNS remained associated with lower PANAS Negative Affect than sham stimulation, Treatment Group: F(1, 35.25) = 5.81, *p* = 0.021, with a significant main effect of Phase, F(3, 93.78) = 22.56, *p* < 0.001, and no significant Treatment Group × Phase interaction, F(3, 93.78) = 0.75, *p* = 0.526. Recent nicotine use was not associated with PANAS Negative Affect, F(1, 35.51) = 0.69, *p* = 0.413.

## Discussion

The present study tested whether acute vagal-afferent modulation with active taVNS, relative to sham stimulation, would be associated with lower self-reported negative affect during a video-based mood-induction paradigm in women with BPD. Based on the interoceptive-threshold framework, we hypothesized that active taVNS would reduce subjective negative affect and explored whether this effect was accompanied by changes in peripheral autonomic indices.

Consistent with the primary hypothesis, active taVNS was associated with lower self-reported negative affect across the mood-induction paradigm after adjustment for baseline negative affect and stimulation intensity. Negative affect varied significantly across task phases, increasing after the negative video blocks and decreasing during recovery, confirming that the mood-induction procedure successfully elicited self-reported negative affect. The Treatment Group × Phase interaction was not significant, indicating that the treatment-group difference was not confined to a specific task phase. Instead, active stimulation was associated with lower overall PANAS Negative Affect across the paradigm. Exploratory physiological analyses showed no evidence of treatment-related effects on heart rate, lnRMSSD, or phasic EDA response frequency. An additional exploratory tonic SCL analysis showed no main effect of Treatment Group, but did show an Epoch effect and a Treatment Group × Epoch interaction.

The present single-session design was intended to examine whether acute vagal-afferent modulation is associated with changes in subjective emotional responding while stimulation is ongoing. This question is distinct from repeated-session neuromodulation protocols aimed at inducing longer-lasting clinical or network-level changes [47]. The observed treatment-group difference should therefore not be interpreted as evidence of durable clinical improvement. Rather, it suggests that active taVNS may acutely influence subjective negative affect during emotional challenge. This interpretation is consistent with the possibility that vagal-afferent stimulation may modulate interoceptive-affective processing, but the present study cannot establish this mechanism directly.

The findings also help refine the role of the interoceptive-threshold framework in this manuscript. This framework was presented as a conceptual rationale derived from Linehan’s model of emotional vulnerability and contemporary interoceptive accounts of emotion, rather than as a fully tested explanatory model. The present results do not demonstrate that taVNS changed interoceptive thresholds, nor do they establish that altered bodily signal processing mediated the reduction in self-reported negative affect. However, the pattern of findings is compatible with the broader idea that subjective emotional distress in BPD may not map directly onto the magnitude of peripheral autonomic responses. In particular, the reduction in self-reported negative affect was not accompanied by corresponding reductions in heart rate, lnRMSSD, or phasic EDA response frequency. This dissociation is consistent with the possibility that acute taVNS may influence how bodily signals are integrated into subjective emotional experience, rather than simply reducing peripheral autonomic activation. Future studies incorporating direct measures of interoceptive accuracy, sensibility, and neural interoceptive processing will be needed to test this mechanism more directly.

A conceptual framework for this dissociation can be drawn from precision-weighted predictive coding accounts of interoception, in which emotional experience depends not only on the magnitude of ascending bodily signals but on the reliability weight assigned to them [15, 16, 56]. In this view, the core disturbance may lie in how prediction errors about internal states are weighted relative to top-down priors, rather than in autonomic output alone [11, 56]. Vagal-afferent stimulation has been proposed to modulate precision parameters within the insula-anterior cingulate hierarchy, potentially recalibrating the balance between interoceptive and exteroceptive inference [12, 38, 39, 56]. The present finding that active taVNS was associated with lower subjective negative affect without corresponding treatment-related effects on heart rate, lnRMSSD, or phasic EDA response frequency, is compatible with this possibility, although the present study cannot test it directly.

The exploratory tonic SCL analysis adds nuance to this interpretation. Tonic SCL showed a significant effect of Epoch, suggesting that the mood-induction paradigm elicited phase-dependent changes not only in subjective negative affect, but also in tonic sympathetic arousal. In addition, the Treatment Group × Epoch interaction suggested that active taVNS may have altered the autonomic response profile across the task, with higher tonic SCL during negative-video exposure in the active group. This pattern does not support a simple peripheral calming effect of taVNS. Rather, one cautious interpretation is that active taVNS may have increased physiological engagement with the emotional challenge while subjective negative affect was lower. This apparent dissociation between subjective and physiological indices is consistent with broader evidence that emotional disturbance in BPD does not map directly onto the magnitude of peripheral autonomic responses [9, 20, 25, 57]. However, this interpretation remains preliminary, because tonic SCL was analyzed as an exploratory outcome and required outcome-specific signal-quality exclusion.

To our knowledge, this is the first study to examine taVNS in patients with BPD. Previous experimental taVNS studies have largely been conducted in healthy participants or non-BPD samples and have suggested possible effects on emotion recognition [42], mood [45], stress-related responding [46], and interoceptive processing [31, 38, 39], although autonomic findings have been inconsistent [41]. The present study extends this literature to a BPD sample and suggests that acute taVNS may be associated with lower subjective negative affect during mood induction. At the same time, the absence of treatment-related effects on peripheral physiological indices indicates that the self-report finding was not accompanied by measurable changes in heart rate, lnRMSSD, or EDA response frequency under the present analytic approach.

More broadly, the present findings fit a literature in which subjective emotional disturbance in BPD is often more consistent and more pronounced than peripheral autonomic abnormalities. Although BPD has long been conceptualized as involving heightened biological emotional vulnerability [7, 8], empirical studies have produced mixed physiological findings, with some reporting reduced heart rate variability, altered resting arousal, or delayed recovery, but less consistent evidence for exaggerated peripheral reactivity during emotional challenge [9, 20, 25–29, 57]. This heterogeneity has led to growing interest in the possibility that the core disturbance may lie not simply in the magnitude of bodily responses, but in how bodily signals are represented, weighted, and experienced [11, 15, 17]. From this perspective, the present pattern of lower subjective negative affect without corresponding treatment-related effects on most peripheral autonomic indices does not necessarily weaken the interoceptive account. Rather, it is compatible with the idea that emotional vulnerability in BPD may reflect altered mapping between bodily fluctuations and subjective affective salience [17, 57].

Several limitations should be considered. First, the study used a single-session design, which precludes conclusions about durability, clinical efficacy, or longer-term symptom change. Second, the sample was modest in size and restricted to women, which improved clinical homogeneity but limits generalizability to broader BPD populations. Third, no healthy control group or clinical control group was included. The absence of a healthy control group limits conclusions about whether the observed pattern is specific to BPD or whether the video material would elicit comparable levels of negative affect in healthy participants. Similarly, the absence of a clinical comparison group limits conclusions about whether the observed effect reflects BPD-specific mechanisms or more general transdiagnostic processes. Although the mood-induction paradigm had been used previously and was empirically shown to elicit negative affect in BPD and comparison samples, the individual video clips were not selected from a single standardized stimulus database with uniform normative valence and arousal ratings. In the present study, the paradigm was used as a within-sample emotional challenge during ongoing active or sham stimulation, rather than to determine whether the stimuli elicit BPD-specific emotional responses. Accordingly, the findings should be interpreted as responses to this specific video-based mood-induction paradigm rather than to standardized affective stimuli more broadly. Fourth, no direct measure of interoception was included; accordingly, the proposed interoceptive-affective mechanism remains inferential rather than directly tested. Fifth, participants were a clinically complex and largely medicated outpatient sample, and concomitant medication use and psychiatric comorbidity may have contributed additional variance to subjective and physiological responses. Sixth, blinding success was not formally assessed, and partial unblinding cannot be excluded given the different electrode locations used in the active and sham conditions; however, adverse events were monitored by a physician present throughout the procedure who inquired about side effects at study completion. Finally, menstrual cycle phase was not assessed or controlled for, despite its potential relevance for autonomic measures in female samples.

Several directions for future research follow from these findings. First, repeated-session studies are needed to determine whether the acute reduction in subjective negative affect observed here can be extended over time and whether it translates into clinically meaningful changes in emotional vulnerability or emotion regulation in BPD. Second, future work should incorporate direct measures of interoception, including behavioral accuracy tasks, self-report questionnaires such as the Multidimensional Assessment of Interoceptive Awareness [12, 58], and neurophysiological indices such as heartbeat-evoked potentials [22, 31], to test whether taVNS-related changes in emotional responding are accompanied by measurable changes in body-brain signal processing. Third, mediation analyses examining whether changes in interoceptive accuracy mediate the relationship between taVNS and emotional responding would directly test this proposed interoceptive-affective mechanism. Fourth, studies including both healthy and clinical comparison groups will be important to determine whether the observed effects are specific to BPD or reflect broader transdiagnostic mechanisms. Finally, future studies should optimize physiological acquisition and analysis strategies, including time-resolved analyses during video presentation and validated decomposition of tonic and phasic electrodermal activity.

## Conclusions

A single session of active taVNS was associated with lower self-reported negative affect across a video-based mood-induction paradigm in women with BPD, whereas exploratory physiological analyses did not provide evidence of treatment-related modulation of heart rate, lnRMSSD, or phasic EDA response frequency. This pattern is consistent with the possibility that acute vagal-afferent stimulation may influence subjective affective responding without producing a uniform peripheral autonomic effect, although the present design cannot establish the underlying mechanism. Future studies incorporating direct measures of interoceptive processing are needed to test this interpretation. These findings are preliminary and should not be interpreted as evidence of durable clinical efficacy. They support further investigation of taVNS as a mechanistic probe of acute affective responding in BPD and may inform future repeated-session studies evaluating taVNS as a potential adjunctive intervention.

## Electronic supplementary material

Below is the link to the electronic supplementary material.

Supplementary material 1

## Data Availability

The datasets generated and/or analysed during the current study are not publicly available due to ethical and privacy restrictions but are available from the corresponding author on reasonable request, subject to approval by the relevant ethics committee and data protection regulations.

## Abbreviations

BPD: Borderline personality disorder
BSL-23: Borderline Symptom List-23
DBT: Dialectical Behaviour Therapy
DERS: Difficulties in Emotion Regulation Scale
ECG: Electrocardiography
EDA: Electrodermal activity
GAD: Generalized anxiety disorder
GSR: Galvanic skin response
HRV: Heart rate variability
PANAS: Positive and Negative Affect Schedule
RMSSD: Root mean square of successive differences
SCL: Skin conductance level
taVNS: Transcutaneous auricular vagus nerve stimulation
