# Supplementary material for: Transcutaneous auricular vagus nerve stimulation and emotional responding in borderline personality disorder: a randomized single-blind, sham-controlled study
Source: Borderline Personal Disord Emot Dysregul. 2026 Aug 4;13:24. doi: 10.1186/s40479-026-00367-x (PMC13435753; doi:10.1186/s40479-026-00367-x)
Supplement: Supplementary file 1 [file 40479_2026_367_MOESM1_ESM.docx]

| **Supplementary Table S1. Internal consistency of self-report measures used in the present sample** | | | | | |
| --- | --- | --- | --- | --- | --- |
| **Measure** | **Scale** | **Items** | **Previously reported internal consistency** | **Valid N** | **Cronbach’s α in present sample** |
| PANAS | Negative Affect, baseline | 10 | α = .84–.87 | 33 | .841 |
| DERS | Total score, corrected after reverse-scoring | 36 | α = .93 | 32 | .898 |
| BSL-23 | Total score | 23 | α = .935–.969 | 34 | .905 |
|  |  |  |  |  |  |
| **Note.** PANAS = Positive and Negative Affect Schedule; DERS = Difficulties in Emotion Regulation Scale; BSL-23 = Borderline Symptom List, 23-item version. Cronbach’s alpha values were calculated using available complete cases for each scale. For the DERS-36, reverse-scored items were recoded before estimating internal consistency. | | | | | |

| **Supplementary Table S2. Prespecified outcomes and analytic implementation** | | | | |
| --- | --- | --- | --- | --- |
|  |  |  |  |  |
| **Prespecified outcome or analysis** | **Registered description** | **Analytic implementation in the present study** | **Analytic clarification** | **Rationale** |
| Primary PANAS post-induction outcome | Change in negative emotional arousal from baseline immediately after mood induction, assessed with PANAS Negative Affect. This corresponded to post-video PANAS Negative Affect ratings obtained after the mood-induction videos. | The primary PANAS model analyzed PANAS Negative Affect across four collapsed task phases: Pre-Induction, Post-Neutral, Post-Negative, and Recovery-Negative. The registered post-induction outcome was also analyzed separately across VideoType and is reported in Supplementary Table S3. | Post-Negative was computed as the mean of the three post-video ratings following the negative videos. Recovery-Negative was computed as the mean of the three recovery ratings following the negative videos. | The joint phase-based model provides a parsimonious estimate of treatment-group differences across the mood-induction paradigm while retaining the registered post-induction outcome in outcome-specific analyses. |
|  |  |  |  |  |
| Pre-induction PANAS Negative Affect | PANAS Negative Affect before mood induction. | Pre-induction PANAS Negative Affect was included in the primary joint PANAS model as the Pre-Induction phase and was also analyzed separately as a registered outcome-specific ANCOVA in Supplementary Table S3. | Pre-Induction refers to ratings obtained after the initial stimulation-alone period and before the video-based mood-induction phase. | This preserves the registered pre-induction outcome while clarifying its timing within the experimental sequence. |
|  |  |  |  |  |
| Recovery PANAS Negative Affect | PANAS Negative Affect during recovery after mood induction. | Recovery PANAS Negative Affect was included in the primary joint PANAS model as Recovery-Negative and was also analyzed separately using post-induction and recovery ratings across the three negative videos in Supplementary Table S3. | Recovery-Negative was computed as the mean of the three recovery ratings obtained 4 min after each negative video. There was no recovery period after the neutral video. | This preserves the registered recovery outcome while providing a phase-level estimate of recovery-related negative affect after negative mood induction. |
|  |  |  |  |  |
| Perceived difficulty managing emotional responses | Perceived difficulty in managing emotional responses during video presentation. | This measure was analyzed separately in Supplementary Table S3 using a mixed-effects model with Treatment Group, VideoType, Treatment Group × VideoType, and stimulation intensity. | This measure was not included in the primary PANAS model because it assesses perceived difficulty managing emotional responses rather than PANAS Negative Affect. | This allows the registered measure to be reported while keeping the primary model focused on self-reported negative affect. |
|  |  |  |  |  |
| Physiological outcomes | ECG- and GSR-derived autonomic indices during the experimental paradigm. | Physiological outcomes were analyzed using baseline-adjusted mixed-effects models across task-defined stimulation and video-viewing epochs: stimulation-alone, neutral-video, and negative-video. The negative-video epoch was computed as the mean across the three negative-video segments. | Physiological epoching was defined at the level of stimulation and video-viewing periods. Baseline physiological values were included as covariates to account for individual differences in resting physiology. | This approach provides a parsimonious estimate of treatment-related differences in physiological responses during stimulation and video viewing while accounting for individual baseline differences. |
|  |  |  |  |  |
| Sample size and exclusions | Target sample size of 42 participants, corresponding to 21 participants per arm, including an expected attrition allowance. Minimum sample size required by the original power calculation was 34 participants. | Thirty-four participants were randomized and included in the primary self-report analyses. Outcome-specific exclusions were applied to physiological analyses based on signal quality: one participant was excluded from ECG-derived analyses because of invalid ECG-derived indices, and one participant was excluded from GSR-based analyses because of implausibly high and invariant tonic SCL values with no detected phasic electrodermal responses. | The final randomized sample reflected the number of eligible participants available within the recruitment period. No randomized participant was excluded from the primary self-report analyses. | This clarifies the relation between the target sample, minimum required sample, final randomized sample, and analysis-specific exclusions. |
|  |  |  |  |  |
| Baseline characteristics | Demographic, clinical, stimulation-related, and physiological baseline characteristics were collected before the experimental procedure. | Baseline characteristics are presented descriptively in Table 1. Significance tests of baseline group differences are not reported. | Baseline characteristics are summarized by randomized group without inferential testing. | This follows CONSORT recommendations for randomized trials, where baseline significance tests are not informative because allocation is by chance. |
| **Note.** PANAS = Positive and Negative Affect Schedule; ECG = electrocardiography; GSR = galvanic skin response; EDA = electrodermal activity. This table describes the implementation of prespecified outcomes and related analytic decisions in the present study. | | | | |

| **Supplementary Table S3. Registered outcome-specific analyses** | | |  |  |  |
| --- | --- | --- | --- | --- | --- |
|  |  |  |  |  |  |
| **Registered outcome** | **Model** | **Treatment-related result** | **Approx. partial η²** | **Other relevant effects** | **Interpretation** |
| Post-induction PANAS Negative Affect | Mixed-effects model with Treatment Group, VideoType, Treatment Group × VideoType, baseline PANAS Negative Affect, and stimulation intensity | Treatment Group: F(1, 34.50) = 2.46, p = .126 | .07 | VideoType: F(3, 90.07) = 57.33, p < .001, ηp² = .66; Treatment Group × VideoType: F(3, 90.07) = 0.53, p = .664, ηp² = .02 | Lower adjusted post-video PANAS Negative Affect in the active taVNS group than in the sham group, but the difference did not reach statistical significance |
| Pre-induction PANAS Negative Affect | ANCOVA with Treatment Group, baseline PANAS Negative Affect, and stimulation intensity | Treatment Group: F(1, 30) = 4.50, p = .042 | .13 | Baseline PANAS Negative Affect: F(1, 30) = 41.45, p < .001, ηp² = .58; stimulation intensity: F(1, 30) = 0.64, p = .430, ηp² = .02 | Active taVNS group showed lower pre-induction PANAS Negative Affect after adjustment for baseline PANAS Negative Affect and stimulation intensity |
| Recovery PANAS Negative Affect | Mixed-effects model with Treatment Group, Time, Treatment Group × Time, VideoType, baseline PANAS Negative Affect, and stimulation intensity | Treatment Group: F(1, 95.00) = 11.35, p = .001; Treatment Group × Time: F(1, 99.00) = 4.30, p = .041 | .11 for Treatment Group; .04 for Treatment Group × Time | Time: F(1, 99.00) = 65.91, p < .001, ηp² = .40; VideoType: F(2, 95.00) = 14.11, p < .001, ηp² = .23 | Larger decrease from post-induction to recovery in the active taVNS group than in the sham group |
| Perceived difficulty managing emotional responses | Mixed-effects model with Treatment Group, VideoType, Treatment Group × VideoType, and stimulation intensity | Treatment Group: F(1, 38.81) = 0.03, p = .868 | < .01 | VideoType: F(3, 90.59) = 21.35, p < .001, ηp² = .41; Treatment Group × VideoType: F(3, 90.59) = 0.24, p = .866, ηp² = .01 | No evidence of a treatment-group difference in perceived difficulty managing emotional responses |
| **Note.** PANAS = Positive and Negative Affect Schedule. For post-induction PANAS and perceived difficulty managing emotional responses, one observation was missing for participant 003 during the domestic violence video. For recovery PANAS, participant 003 had missing values for both post-induction and recovery ratings for the domestic violence video. Mixed-effects models used all available observations without manual imputation. Sensitivity analyses additionally adjusting for VideoOrder yielded the same pattern of findings. Approximate partial η² values were calculated from the F statistic and denominator degrees of freedom. | | | | | |

| **Supplementary Table S4A. Exploratory secondary physiological model tests** | | | | | | | |
| --- | --- | --- | --- | --- | --- | --- | --- |
|  |  |  |  |  |  |  |  |
| **Outcome** | **Effect** | **Active taVNS adjusted mean** | **Sham stimulation adjusted mean** | **F(df1, df2)** | **p** | **Approx. partial η²** | **Interpretation** |
| Heart rate, bpm | Treatment Group | 76.92 | 78.13 | F(1, 29.38) = 1.32 | .260 | .04 | No evidence of a treatment-group difference |
| Heart rate, bpm | Epoch | — | — | F(2, 60.44) = 0.73 | .485 | .02 | No significant epoch effect |
| Heart rate, bpm | Treatment Group × Epoch | — | — | F(2, 60.44) = 0.04 | .965 | < .01 | No evidence of a treatment-group difference varying across epochs |
| Heart rate, bpm | Baseline heart rate | — | — | F(1, 29.45) = 462.25 | < .001 | .94 | Baseline heart rate strongly predicted task heart rate |
| Heart rate, bpm | Stimulation intensity | — | — | F(1, 29.45) = 0.24 | .631 | .01 | Not significant |
| Heart rate, bpm | Recent nicotine use | — | — | F(1, 29.45) = 0.12 | .736 | < .01 | Not significant |
| lnRMSSD | Treatment Group | 3.56 | 3.43 | F(1, 33.69) = 1.84 | .184 | .05 | No evidence of a treatment-group difference |
| lnRMSSD | Epoch | — | — | F(2, 64.15) = 0.86 | .430 | .03 | No significant epoch effect |
| lnRMSSD | Treatment Group × Epoch | — | — | F(2, 64.15) = 0.41 | .663 | .01 | No evidence of a treatment-group difference varying across epochs |
| lnRMSSD | Baseline lnRMSSD | — | — | F(1, 34.03) = 123.69 | < .001 | .78 | Baseline lnRMSSD strongly predicted task lnRMSSD |
| lnRMSSD | Stimulation intensity | — | — | F(1, 34.03) = 0.53 | .471 | .02 | Not significant |
| lnRMSSD | Recent nicotine use | — | — | F(1, 34.03) = 1.38 | .248 | .04 | Not significant |
| EDA peaks/min | Treatment Group | 3.36 | 2.70 | F(1, 31.62) = 1.75 | .195 | .05 | No evidence of a treatment-group difference |
| EDA peaks/min | Epoch | — | — | F(2, 61.91) = 0.95 | .393 | .03 | No significant epoch effect |
| EDA peaks/min | Treatment Group × Epoch | — | — | F(2, 61.91) = 0.06 | .945 | < .01 | No evidence of a treatment-group difference varying across epochs |
| EDA peaks/min | Baseline peaks per minute | — | — | F(1, 32.07) = 99.67 | < .001 | .76 | Baseline peaks per minute strongly predicted task peaks per minute |
| EDA peaks/min | Stimulation intensity | — | — | F(1, 32.07) = 0.34 | .562 | .01 | Not significant |
| EDA peaks/min | Recent nicotine use | — | — | F(1, 32.07) = 0.92 | .344 | .03 | Not significant |
| Tonic SCL, µS | Treatment Group | 3.36 | 3.11 | F(1, 28.07) = 0.41 | .525 | .01 | No evidence of a treatment-group difference |
| Tonic SCL, µS | Epoch | — | — | F(2, 61.38) = 4.59 | .014 | .13 | Significant epoch effect |
| Tonic SCL, µS | Treatment Group × Epoch | — | — | F(2, 61.38) = 4.86 | .011 | .14 | Exploratory interaction; interpreted cautiously |
| Tonic SCL, µS | Baseline tonic SCL | — | — | F(1, 27.73) = 206.36 | < .001 | .88 | Baseline tonic SCL strongly predicted task tonic SCL |
| Tonic SCL, µS | Stimulation intensity | — | — | F(1, 27.73) = 0.32 | .578 | .01 | Not significant |
| Tonic SCL, µS | Recent nicotine use | — | — | F(1, 27.73) = 1.97 | .172 | .07 | Not significant |
| **Note.** EDA = electrodermal activity; EDA peaks/min = phasic EDA response frequency indexed as peaks per minute; lnRMSSD = natural-log-transformed root mean square of successive differences; SCL = skin conductance level. Models were estimated across task-defined stimulation and video-viewing epochs: stimulation-alone, neutral-video, and negative-video. The negative-video epoch was computed as the mean across the three 4-min negative video segments. The corresponding baseline physiological value, stimulation intensity, and recent nicotine use were included as covariates in each model. Recent nicotine use was defined as any nicotine use during the 12 h preceding the experiment. Adjusted means are estimated marginal means for Treatment Group. Epoch-specific and Treatment Group × Epoch estimated marginal means are reported in Supplementary Table S4B. Approximate partial η² values were calculated from the F statistic and denominator degrees of freedom. Outcome-specific exclusions were applied based on signal quality: one participant was excluded from ECG-derived analyses because of invalid ECG-derived indices, and one participant was excluded from GSR-based analyses because of implausibly high and invariant tonic SCL values with no detected phasic electrodermal responses. | | | | | | | |

| **Supplementary Table S4B. Estimated marginal means for exploratory physiological outcomes by epoch and treatment group** | | | | |
| --- | --- | --- | --- | --- |
| **Outcome** | **Epoch** | **Overall adjusted mean [95% CI]** | **Active taVNS adjusted mean [95% CI]** | **Sham stimulation adjusted mean [95% CI]** |
| Heart rate, bpm | Stimulation-alone | 77.35 [76.06, 78.64] | 76.70 [74.87, 78.53] | 78.00 [76.23, 79.78] |
| Heart rate, bpm | Neutral-video | 77.87 [76.58, 79.15] | 77.22 [75.39, 79.05] | 78.51 [76.74, 80.29] |
| Heart rate, bpm | Negative-video | 77.35 [76.07, 78.64] | 76.85 [75.02, 78.68] | 77.86 [76.09, 79.64] |
| lnRMSSD | Stimulation-alone | 3.51 [3.38, 3.63] | 3.54 [3.37, 3.71] | 3.47 [3.30, 3.64] |
| lnRMSSD | Neutral-video | 3.52 [3.40, 3.64] | 3.59 [3.41, 3.76] | 3.46 [3.29, 3.63] |
| lnRMSSD | Negative-video | 3.45 [3.33, 3.57] | 3.55 [3.37, 3.72] | 3.35 [3.18, 3.52] |
| EDA peaks/min | Stimulation-alone | 3.11 [2.55, 3.68] | 3.50 [2.66, 4.35] | 2.72 [1.90, 3.54] |
| EDA peaks/min | Neutral-video | 3.19 [2.62, 3.75] | 3.51 [2.66, 4.35] | 2.87 [2.05, 3.69] |
| EDA peaks/min | Negative-video | 2.79 [2.22, 3.36] | 3.06 [2.22, 3.91] | 2.52 [1.69, 3.34] |
| Tonic SCL, µS | Stimulation-alone | 3.09 [2.70, 3.48] | 3.11 [2.53, 3.70] | 3.07 [2.50, 3.63] |
| Tonic SCL, µS | Neutral-video | 3.19 [2.80, 3.58] | 3.24 [2.65, 3.82] | 3.14 [2.58, 3.71] |
| Tonic SCL, µS | Negative-video | 3.43 [3.04, 3.82] | 3.74 [3.16, 4.33] | 3.11 [2.55, 3.68] |
| **Note.** Note. EDA = electrodermal activity; EDA peaks/min = phasic EDA response frequency indexed as peaks per minute; lnRMSSD = natural-log-transformed root mean square of successive differences; SCL = skin conductance level. Values are estimated marginal means from the same mixed-effects models reported in Supplementary Table S4A. Overall adjusted means are marginal means across treatment groups. Treatment-group adjusted means are shown within each task-defined epoch. Stimulation-alone refers to the initial stimulation-only epoch before video presentation. The negative-video epoch was computed as the mean across the three 4-min negative video segments. | | | | |
